# The Role of Minimally Invasive Spinal Surgical Procedures in the Elderly Patient: An Analysis of 49 Patients Between 75 and 95 Years of Age

**DOI:** 10.7759/cureus.7180

**Published:** 2020-03-04

**Authors:** Michael Warhurst, Jason Hartman, Michelle Granville, Robert E Jacobson

**Affiliations:** 1 Pain Medicine, Larkin Community Hospital, Miami, USA; 2 Pain Medicine, The Spine and Orthopedic Center, Santa Barbara, USA; 3 Neurosurgery, University of Miami Hospital, Miami, USA

**Keywords:** neurogenic claudication, osteoporotic vertebral fractures

## Abstract

As the population gets older, yet remains active, the number of patients presenting with symptomatic spinal disease over the age of 75 increases. These include pain from osteoporotic spinal fractures, lumbar degenerative disease, as well as radiculopathy or neurogenic claudication from stenosis over the age of 75 and older increases. While some of these patients are very healthy, taking minimal medication, many are not good candidates for more invasive surgical procedures under general anesthesia because of medical co-morbidities such as insulin-dependent diabetes and medication use such as anti-coagulants. Past reviews of lumbar surgery in elderly patients have examined the risk factors with spinal fusion and multilevel surgery and many were written before the recent advent of more minimally invasive spinal procedures that reduce both operative time and the need for general anesthesia. This review examines effectiveness in return to activity and reduction in pain in these elderly patients stratified by underlying disease category, i.e. fractures, stenosis with neurogenic claudication and chronic pain, rather than just by the procedure, since there are often several minimally invasive procedures that are available. This review demonstrates very similar pain relief outcomes as measured by the visual analog scale (VAS) scores which dropped in the range of 70% or more with the different procedures. Since the majority of these procedures involve short surgical times and minimal blood loss with small incisions that lower the risk of wound infection as well as cardio-respiratory stress and can be performed under local anesthesia as an outpatient, they are particularly advantageous for the properly selected elderly patient.

## Introduction

The perception of how old is 'elderly' and what spinal surgical procedures can be safely performed on these patients has changed over time. In the 1960s and 1970s, early articles regarding elderly lumbar spine surgery defined elderly as age 65 while later articles gradually expanded the range from 75 to 80 to over 90 years of age [1-4]. It is common now to see patients in their 80s and even 90s who have been active, but in a relatively short time became incapacitated from acutely symptomatic spinal problems either because of acute pain or restriction of activity, especially walking due to radiculopathy or neurogenic claudication [5]. The use of both magnetic resonance imaging (MRI) and computed tomography (CT) make accurate diagnosis possible in these patients allowing better localization of the spinal pathology. A subgroup of these patients is very active and healthy using minimal medications, while others have a multitude of medical problems that make prolonged general anesthesia, surgery and recovery and rehabilitation difficult if not impossible. Patients are generally risk stratified by using the American Society of Anesthesiology (ASA) guidelines [6-7]. However, there are additional indexes such as the Frailty Index and the Charlson Comorbidity Index (CCI) that consider associated medical issues coincident with age [8-12]. 

When assessing 'elderly' patients, there is a distinct group of patients with symptomatic severe spinal stenosis, spinal instability or deformity who are best treated with open multilevel surgical procedures if they meet anesthesia and surgical risk profiles [12-16]. Besides general medical anesthesia risks, studies have identified high-risk spinal surgery patients based on elevated baseline metabolic index (BMI), insulin-dependent diabetes, preoperative anticoagulant use, excessive blood loss, and prolonged operative time [13-15]. In the elderly patient, mental status, the presence of dementia and Alzheimer's disease, as well as Parkinson's Disease add additional peri-operative and intra-operative risk factors for general anesthesia but even local anesthesia with sedation [16-17]. There is also another group of elderly patients who either do not want extensive open surgery or are at higher risk and may be more suitable for more minimally invasive procedures with the caveat that if symptoms do not sufficiently improve there is the option to still proceed with more extensive surgery [18]. Balancing all these factors, patients in the 80s and 90s can still undergo different levels of extensive lumbar decompression and fusion with no significantly greater peri-operative risk and acceptable outcomes [19-20]. Over the last 10 or more years, minimally invasive spinal procedures have evolved, which combined with the use of local anesthesia enable surgery to be performed as an outpatient and allowing quicker reduction of pain and return to full activity. In healthy patients, this allows them to continue their lifestyle while patients regarded to be at very high risk for more complicated spinal procedures and especially general anesthesia can get pain relief, improvement in activity and quality of life due to relief of neurogenic claudication very similar to more complicated procedures such as decompression and fusions with or without screw fixation [21-23]. The most common spinal problems that are seen in the elderly population and in which minimally invasive spinal procedures are applicable include vertebral compression fractures with acute incapacitating or later chronic back pain, spinal stenosis with neurogenic claudication, acute far lateral discs that are often seen in the upper lumbar spine, and chronic pain secondary to failed laminectomy, degenerative scoliosis, and spondylosis often in combination with diabetic or vascular neuropathy [23-24]. As the spinal surgeon, interventional pain management specialist or even interventional radiologists and especially anesthesia team become comfortable with performing procedures under local anesthesia, and as the operative time of the experienced surgeon becomes shorter, it is possible to recommend minimal invasive procedures when supported by diagnostic studies with faster return home, ambulation, post-procedure physical therapy, and less morbidity with similar outcomes [25-26].

## Materials and methods

A retrospective chart review of patients who underwent different minimally invasive lumbar surgical procedures as an outpatient under local anesthesia for spinal osteoporotic compression fractures, spinal stenosis or disc herniation was made. The starting age for the review was 75 and the patients were stratified by age from 75-80, 80-90 and above 90 years of age. All patients undergoing purely pain management injections or radiofrequency rhizotomy procedures were excluded. The pre-procedure activity, Baseline Metabolic Index (BMI), co-morbidities and medications used, type of procedure performed and anesthesia given, length of procedure, and discharge time, as well as the return of activity with pre- and post-visual analog scale (VAS), was tabulated.

Location of surgery and type of anesthesia used: The primary location where surgery was performed was one of several outpatient ambulatory surgical centers using local anesthesia with mild sedation. If the patient had major medical co-morbidities such as cardiac disease including the presence of a cardiac pacemaker or defibrillator or respiratory disease, a hospital outpatient setting was selected. General anesthesia was used if selected by the patient or anesthesiologist based on pre-procedure grading and surgical positioning in the prone position in an obese patient or with chronic respiratory disease. The protocol for giving local anesthesia, including the use of lidocaine 1% without epinephrine for skin and superficial fascia was used. Following this, 0.5% bupivacaine without epinephrine was given for deeper fascia and adjacent to the periosteum of the bone in the area of the surgery. At least five minutes was given to provide time for local anesthesia to work and at time a mixture of 1/4 lidocaine and 3/4 bupivacaine was used. At the end of all procedures, additional bupivacaine was injected through all access cannulas before being withdrawn. In patients undergoing CoflexR (RTI Surgical, Alachua, Florida, USA) procedures, the fascia and deep muscle were also injected with a long-acting lysosomal preparation of 0.2% bupivacaine diluted in a 50% dilution with normal saline.

Patient classification for this review: Previously anesthesia grades have been well established in pre-surgical risk evaluation. We decided to categorize patients by VAS pain level combined with pre-operative activity and known morbidities. Group A was a previously healthy patient with full activity and no medical or anesthesia restrictions, group B if the moderately active patient with multiple medical co-morbidities and/or medications that may be problematic for general anesthesia or surgery, and group C is a patient with a previously restricted lifestyle and multiple medical comorbidities.

Patients with osteoporotic compression fractures ranged from active patients that had a sudden fall or injury who developed an acute osteoporotic compression fracture to those that had moderate pre-injury restrictions, often a history of osteoporosis with previous fractures, either in the vertebral column or long bones and another group with an acute fracture but combined with various co-morbidities such as dementia, diabetes, and cardiac or pulmonary disease, or chronic steroid use that made them both a poor anesthesia risk as well as surgical risk. Many have either cardiac pacemakers, defibrillators or are on anti-coagulants. Procedures performed ranged from unilateral or bilateral vertebroplasty with vertebral augmentation, balloon kyphoplasty and SpineJack^R ^implants with height restoration (Stryker Corporation, Kalamazoo, Michigan, USA). ^ ^Procedures were also classified if 1 or multiple spinal levels were treated.

Patients with lumbar spinal stenosis with neurogenic claudication ranged from active, healthy patients with at least 6 months or greater development of progressive restrictions of walking that are now affecting normal activity. They had a confirmatory high score on the Zurich Claudication Index (ZCI), lower Oswestry Disability Index (ODI) with radiologic studies including CT and MRI showing either one level or two level stenosis rather than diffuse multilevel stenosis. Within this group, many had medical co-morbidities with the use of medications such as anticoagulants or had more chronic restrictions that have progressed to total interference with daily activities with the use of a walker or wheelchair because of pain and inability to walk. There are multiple applicable minimally invasive procedures depending on the clinical and anatomic/radiologic severity of the stenosis. These range from minimally invasive lumbar decompression procedure (MILD) (Vertos Medical, Aliso Viejo, California, USA), interspinous stabilization with VertiflexR interspinous spacer(Superion Corporation, San Clemente, California, USA) and localized lumbar decompression with CoflexR interlaminar implant instead of extensive laminectomy with or without instrumentation.

Chronic spinal and /or limb pain patients included pain patients that have not obtained lasting relief with various facet, epidural or foraminal injections or subsequent radiofrequency rhizotomy. Despite advanced age, they had undergone an initial spinal cord stimulation trial placement of an epidural electrode for pain control with a 5 to 7 day testing period and if successful with at least 50% pain relief they would be candidates for permanent implantation of epidural electrodes and battery. In all our patients, after the initial trial, rather than inserting a permanent paddle lead through a thoracic laminotomy, these patients were offered implantation of one or two percutaneous leads with a battery placed under local anesthesia.

## Results

There were a total of 49 patients in all three groups over 15 months. There were 22 patients with osteoporotic compression fractures, 18 with lumbar stenosis and neurogenic claudication and nine with intractable chronic back and lower extremity pain. The average age for all categories was 82.3 and BMI 27.8 of all groups. There was predominance of females which may be related to the longer life expectancy of females over males. There were 24 patients on low-dose 81 mg aspirin and seven on various anticoagulants before surgery. If on anticoagulants, the patient had medical clearance that anticoagulants could be stopped and held for a minimum of 3 days after the procedure and then were ceased for the recommended period of 2 to 7 days depending on the class of anti-coagulants. There were no cases of wound hematoma or excessive bleeding postoperatively, although several patients had ecchymosis extending in a wider area around the surgical area. There were 28 diabetic patients, 20 on metformin or glipizide, and eight on insulin. The patients on insulin had a medical evaluation and were told to stop or continue partial doses the morning of surgery. All were allowed to take their medication the morning of the procedure. All patients received either 2 g cefoxin or 600 mg cleocin intravenous if allergic to penicillin or the cefoxin group and usually remained on antibiotics for three days post-procedure. There were no wounds or deeper infections post-operatively. The only patients (two of the entire series) undergoing general anesthesia were either by patient choice or anesthesiologist concern of surgery in the prone position primarily with patients with respiratory compromise. Procedures were performed as an outpatient under local anesthesia with monitored anesthesia care (MAC) sedation in 47 of the 49 patients. Two patients had procedures under general anesthesia. Both had stenosis involving lumbar decompression with an interlaminar stabilizing device (Coflex) but were discharged home the same day. In several patients, the procedure was scheduled in the hospital outpatient surgical department rather than a free-standing ambulatory surgery center because of a combination of advanced age usually over 80 with extensive comorbidities, especially the presence of a cardiac pacemaker or defibrillator, or marked respiratory issues. All patients were discharged home within two to four hours after the procedures except one patient, a 90 year old female with severe Alzheimer's disease who had to stay for a 23 hour admission because of prolonged somnolence after MAC anesthesia. Pre-procedure VAS was 8.47, and regardless of procedure performed, the post-procedure score was 70% lower. For the entire group of patients, there was no re-hospitalization, neurologic or wound complications.

Of the 22 fracture patients, 18 were female with an average age of 82.7 years. Five had multiple fractures treated at the same time and 70% had radiologic evidence of previous spinal fractures. Interestingly 17 of the 22, or 77%, were at the thoracic-lumbar junction from T11 to L1. Five of the 22, or 18%, had two acute fractures treated at the time of surgery (Table 1).

**Table 1:** Osteoporotic fractures M = male, F = female, L = lumbar, T= thoracic, V = vertebroplasty, K = kyphoplasty, SJ = kyphoplasty with SpineJack, A = ambulatory center, H = hospital, G = general, M+L = MAC and local, MAC = monitored anesthesia care, VAS = visual analog scale

Sex	Age	Fracture Level	Procedure	Pre-VAS	Post-VAS
F	88	L1, L2	V	10	2
F	98	T10	V	8	2
F	75	L1	V	10	3
M	79	T11, T12	V	10	5
F	90	L1	V	8	4
F	77	T9, T10	V	6	2
F	81	L3	V	6	0
F	91	T8	V	8	2
F	88	L5	K	10	5
F	88	L1	K	5	0
F	79	L1	K	8	0
F	89	L1	K	5	1
F	81	L1	SJ	8	0
M	79	L1	SJ	7	1
F	80	T12	SJ	8	3
F	77	T11	SJ	7	1
F	81	T12	SJ	10	2
F	90	L3, L4	SJ	9	4
F	95	T11, T12	SJ	8	0
M	82	T12	SJ	10	3
M	78	L1	SJ	4	0
F	92	L1	SJ	9	0
Sex Totals	Age Average		Procedure Totals	Pre-VAS Average	Post-VAS Average
F = 18, M = 4	84.45				
F = 7, M = 1	84.88		Vertebroplasty = 8	8.25	2.5
F = 11, M = 3	84.21		Kyphoplasty +/- SpineJack = 14	7.71	1.43

In the 18 lumbar stenosis patients, there were very similar outcomes in return to ambulation and VAS score reduction using either Coflex^R^ or Vertiflex^R^ implants and procedures. The decision to use Coflex^R^ was based on more constant pain not totally relieved with sitting, higher Zurich claudication scores combined with radiologic severity of the stenosis (> 50%) and especially the presence of degenerative spondylolisthesis. The average pre-operative VAS was 8 and again there was a drop to 2.44 or 70%; however; the Coflex^R^ implant patients started with an average higher VAS of 8.88, while the Vertiflex^R^ patients averaged a VAS of 7.6. Interestingly, there were two patients who were initially seen for single osteoporotic fractures and as they resumed activities after kyphoplasty had symptoms of neurogenic claudication. A retrospective review revealed these symptoms were present before the fall leading to the fracture and then subsequently underwent a single or double level stenosis procedure with a Vertiflex^R^ implant (Table 2).

**Table 2:** Stenosis M = male, F = female, L = lumbar, T= thoracic, V = vertiflex, C = coflex, MILD= minimally invasive lumbar decompression, A = ambulatory center, H = hospital outpatient, G = general, M+L = MAC and local, MAC = monitored anesthesia care, VAS = visual analog scale

Age	Sex	Procedure	Location	Anesthesia	Pre-VAS	Post-VAS
87	F	V (2)	A	M+L	10	2
92	F	V	A	M+L	5	1
81	F	V (2)	A	M+L	4	1
80	F	V (2)	A	M+L	10	3
80	F	V	A	M+L	10	2
75	F	V	A	M+L	8	5
79	M	V (2)	A	M+L	8	2
94	F	V (2)	H	M+L	8	1
89	M	V (2)	A	M+L	6	2
77	F	V (2)	A	M+L	7	1
76	F	C	A	M+L	8	3
79	F	C	A	G	10	5
79	F	C (2)	A	M+L	10	3
81	F	C (2)	A	M+L	9	0
90	F	C	H	G	10	4
83	F	C	A	M+L	9	2
78	M	C (2)	A	M+L	7	0
76	F	MILD (2)	A	M+L	8	4
Average Age	Sex Totals	Procedure Totals	Location	Anesthesia	Pre-VAS Average	Post-VAS Average
82	F = 15, M = 3		A = 16, H = 2	M+L = 16, G = 2	8.17	2.28
83.4	F = 8, M = 2	Vertiflex = 10	A = 9, H = 1	M+L = 10, G = 0	7.60	2.00
80.86	F = 6, M = 1	Coflex = 7	A = 6, H = 1	M+L = 5, G = 2	8.88	2.63
76	F = 1, M = 0	MILD = 1	A = 1, H = 0	M+L = 1, G = 0	8	4

In the nine chronic pain patients, 50% were female and five had chronic post-laminectomy/fusion pain. The remainder had a mixture of diabetic neuropathy, vascular insufficiency pain, and multilevel spinal degeneration with chronic lumbar pain. All had a successful short-term percutaneous trial of five to seven days and then implantation of percutaneous leads with a permanent battery all under local anesthesia as an outpatient. There were no infections, peri-operative complications, or lead migrations (Table 3).

**Table 3:** Pain/spinal cord stimulator M = male, F = female, A = ambulatory center, H = hospital, G = general, M+L = MAC and local, MAC = monitored anesthesia care, VAS = visual analog scale

Age	Sex	Location	Anesthesia	Pre-VAS	Post-VAS
80	F	A	M+L	9	4
83	M	A	M+L	10	2
84	M	H	M+L	6	1
81	F	A	M+L	8	2
83	F	H	M+L	8	4
82	F	A	M+L	7	3
84	M	A	M+L	6	0
84	M	A	M+L	6	0
74	F	A	M+L	7	0
Average Age	Sex Total		Anesthesia	Pre-VAS Average	Post-VAS Average
81.67	F = 5, M = 4		100% M+L	7.44	1.78

## Discussion

Undertaking any form of spinal surgery in the elderly patients must consider their underlying health, comorbidities, presence of dementia and Alzheimer's disease, activity, and pain level. When diagnostic testing after the failure of conservative treatment determines that there is a possibly surgical correctable problem such as an osteoporotic fracture or lumbar stenosis, there are multiple options. However, consideration must be given to the risk of the proposed procedures, type of anesthesia required, surgical time and estimated blood loss [12-14]. The experience of each surgeon with the specific minimally invasive procedure being performed and his/her adeptness at delivering local anesthesia in proper amounts and to the proper location is critical to minimizing the amount of overall sedation the patient receives. All of these factors affect the risk of peri-operative complications that may cause a longer hospital stay and increase the risk of readmission [10,13]. Several large studies have reported complications from fusion ranging from 5.1% to as much as 13% in elderly patients with multilevel surgery and instrumentation [13-14]. The majority of spinal surgery complications are mostly related to excessive blood loss, wound healing, cerebrospinal fluid leakage, hardware misplacement, pulmonary embolism, and thrombophlebitis [6-7,20]. The ASA grade is roughly correlated with the risk of major complications [6-7]. The Frailty Index and the Charlson Co-morbidity Index uses 17 or more sub-factors to estimate the chance of complications in older patients that affect long-term return to functional activity or survival due to age and frailty [11-12]. However, in the elderly, another major concern is the anesthesia effect on unrecognized dementia and cognitive decreases besides what occurs in the more obvious patients with a diagnosis of Alzheimer’s disease [16-17]. In a study of osteoporotic hip fractures, in an aged population, similar to our patients, with osteoporotic vertebral fractures and stenosis, 19.2% under further detailed medical history was found to have dementia and 41.8% some degree of a cognitive disorder [27].

The type of anesthesia used and application for using local anesthesia with mild sedation for minimally invasive procedures is evaluated on at least four main factors: First, the underlying spinal disease process needs to be anatomically very localized. This is based on the radiographic and clinical evaluation to determine if the patient is appropriate for minimally lumbar spinal procedures. Second, the medical status and condition of the patient to determine if they are suitable for the procedure being performed under local anesthesia with sedation: This requires a clear explanation to the patient as well as the patient's clear understanding of the procedure and his/her cooperation during the procedure. Third, the experience of the anesthesiologist using the appropriate amount of moderate sedation anesthesia as well as familiarity with the surgeon and the specific procedure: The anesthesiologist needs to know when and what parts of the procedure require more anesthesia pain control and control of any unintended patient movement. Fourth and finally, the experience of the surgeon with a particular procedure that obviously includes his technical skill and speed as well as the ability to adapt to unusual findings is essential. The decision to perform an even shorter procedure under general anesthesia is usually determined by the anesthesiologist after evaluating the ASA grade, being familiar with the procedure, the surgeon's experience and usual operative time for any specific procedure. However, it must be remembered that elderly patients with dementia can be adversely and sometimes permanently affected by general anesthesia [16-17]. Patients with Parkinson's disease are more prone to falls and spinal fractures and maybe more rigid which may affect surgical positioning [28]. Although extremity tremors may be present, usually this does not interfere with procedures but they may be very sensitive to general anesthesia as well as medication used in light sedation anesthesia [29]. It is important to understand that even mild to moderate sedation used with local anesthesia carries risks, and can lead to post-operative somnolence and cognitive problems in elderly patients. Elderly patients can have various levels of memory loss and in cases with more advanced dementia, they may need prolonged post-procedure recovery or even hospitalization. In our series, there were four patients with varying degrees of Parkinson’s disease and eight with different degrees of dementia. As mentioned in results, one patient, a 90-year-old female, with moderately severe Alzheimer’s dementia and multiple lumbar fractures with severe pain, underwent a two-level bilateral kyphoplasty under local anesthesia with minimal MAC sedation for only 35 minutes and yet was somnolent for four hours after surgery and had to be kept in the hospital for 23 hours observation. Within eight hours after the procedure, she had returned to her pre-procedure mental state. Although, as in this report, the majority of procedures can be performed safely in an ambulatory surgery setting, special attention should be given to the very elderly, especially with medical conditions such as pacemakers, defibrillators, chronic obstructive pulmonary disease (COPD), and respiratory compromise and any degree of dementia. These may be better handled as a 23-hour hospital admission or in a setting that allows prolonged observation.

This small group of very similar elderly patients, based on age and pre-operative VAS scores, reviewed over 15 months shows the very marked improvement in overall VAS and activity possible even with minimal invasive procedures and minimal peri-operative morbidity. There were no deaths, no neurologic complications, no infections, and only one unplanned 23-hour post-procedure admission for post-anesthesia confusion that cleared. Regardless of pathology and procedure, there was a 70+% reduction in VAS and return to significant activity and quality of life for both acute and sub-acute, single or multiple fractures and spinal stenosis with claudication. Continued follow-up, in this series up to 15 months, showed continued long-term improvement in VAS score and mobility and no readmission for further procedures at the surgical site. Generally, the patients who were previously active with minimal use of medications had the best improvement both with either acute or subacute osteoporotic fracture or spinal stenosis with neurogenic claudication many listing their post-procedure VAS as zero. This indicates that when properly selected and performed, these procedures are a reasonable and effective option to more extensive invasive procedures. More open procedures have higher attendant rates of complications and higher rates of anesthesia and peri-operative risks because of a combination of factors including longer operative time, cardiovascular stress secondary to blood loss, risk of neurologic complications, dural tears and possible cerebrospinal fluid leaks, and issues with wound healing in patients on anticoagulants and diabetics.

## Conclusions

With proper evaluation of the elderly patient's spinal symptoms and correlated with CT and MRI findings, combined with assessment of anesthesia and surgical risk and management of peri-operative medicines such as anti-coagulants and diabetes medication, it is possible to perform many different minimally invasive spinal procedures under local anesthesia with minimal risk. Special precautions should be taken with patients with signs of dementia and cognitive disorders both during the procedure and peri-operative period. Post-procedure outcomes were very consistent and surprisingly not dependent on the specific procedure, with a significant decrease in pain and improvement in overall activity at follow-up visit within 30 days of the procedures.
